# Domain Motions and Functionally-Key Residues of l-Alanine Dehydrogenase Revealed by an Elastic Network Model

**DOI:** 10.3390/ijms161226170

**Published:** 2015-12-09

**Authors:** Xing-Yuan Li, Jing-Chao Zhang, Yan-Ying Zhu, Ji-Guo Su

**Affiliations:** College of Science, Yanshan University, Qinhuangdao 066004, China; lxy@ysu.edu.cn (X.-Y.L.); yywlxzyy@163.com (Y.-Y.Z.)

**Keywords:** *Mycobacterium tuberculosis*l-alanine dehydrogenase, domain motions, functionally-key residues, Gaussian network model, anisotropy network model, thermodynamic cycle method

## Abstract

*Mycobacterium tuberculosis* l-alanine dehydrogenase (l-*Mt*AlaDH) plays an important role in catalyzing l-alanine to ammonia and pyruvate, which has been considered to be a potential target for tuberculosis treatment. In the present work, the functional domain motions encoded in the structure of l-*Mt*AlaDH were investigated by using the Gaussian network model (GNM) and the anisotropy network model (ANM). The slowest modes for the open-apo and closed-holo structures of the enzyme show that the domain motions have a common hinge axis centered in residues Met133 and Met301. Accompanying the conformational transition, both the 1,4-dihydronicotinamide adenine dinucleotide (NAD)-binding domain (NBD) and the substrate-binding domain (SBD) move in a highly coupled way. The first three slowest modes of ANM exhibit the open-closed, rotation and twist motions of l-*Mt*AlaDH, respectively. The calculation of the fast modes reveals the residues responsible for the stability of the protein, and some of them are involved in the interaction with the ligand. Then, the functionally-important residues relevant to the binding of the ligand were identified by using a thermodynamic method. Our computational results are consistent with the experimental data, which will help us to understand the physical mechanism for the function of l-*Mt*AlaDH.

## 1. Introduction

Tuberculosis is still a serious threat to human health worldwide, which is caused by the infection of the *Mycobacterium tuberculosis* (*Mt*) [1]. World Health Organization (WHO) has pointed out that at least one-third of the population in the world could be infected with latent *Mt* [2]. Up to now, the treatment of tuberculosis is still a difficult challenge due to the emergence of the drug-resistant strains of *Mt* [3]. At present, it is found that many anti-tubercular drugs become ineffective for the treatment of *Mt*, and thus, the development of new anti-tubercular drugs to treat this disease is urgent [4]. When the pathogens are under nutrient starvation regimes or anaerobic growth conditions, Rv2780 encoding l-alanine dehydrogenase (l-AlaDH) is overexpressed in persistent *Mt* [5]. The production and activity of l-AlaDH were improved when *Mt* was transferred from the aerobic to the anaerobic growth conditions [6]. l-AlaDH can catalyze the 1,4-dihydronicotinamide adenine dinucleotide (NAD)-dependent conversion of l-alanine to ammonia and pyruvate or convert pyruvate to l-alanine in the reverse direction. The catalytic mechanism can be expressed as [7]:

(1) $${\text{L-alanine + NAD}}^{\text{+}}{\text{+ H}}_{2}\text{O}⇌{\text{Pyruvate + NADH + H}}^{\text{+}}{\text{+ NH}}_{\text{4}}^{+}$$

Because the crystallographic structure of human l-AlaDH has not been determined, l-alanine dehydrogenase from *Mycobacterium tuberculosis* (l-*Mt*AlaDH) is studied here. l-*Mt*AlaDH is a secretory antigen, which is closely relevant to bacterial persistence during the persistent stage of infection [8]. Therefore, l-*Mt*AlaDH is considered to be a potential target for the development of anti-tubercular drugs [9].

**Figure 1:**
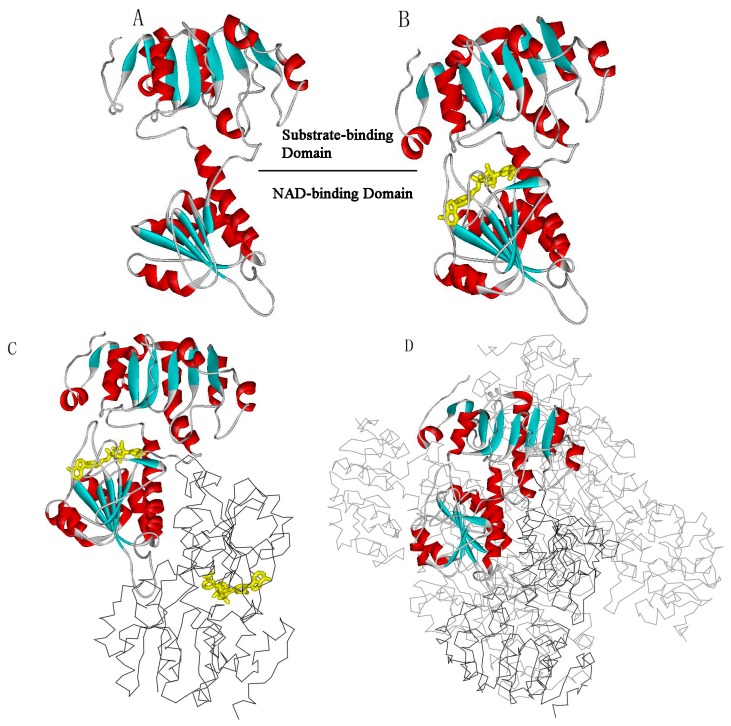
Open-apo (one monomer of 2VOE) (**A**) and closed-holo (one monomer of 2VHZ) (**B**) structures of l-*Mt*AlaDH. The bound ligand is shown as a yellow ball-and-stick model in the closed-holo structure. The secondary substructures α-helices and β-sheets of the protein are displayed in red and blue colors, respectively. The protein is divided into the substrate-binding domain and the 1,4-dihydronicotinamide adenine dinucleotide (NAD)-binding domain, which is marked in the structure; (**C**) The dimeric l-*Mt*AlaDH molecule. One monomer is shown in the solid ribbon model, and the other is shown in gray; (**D**) The hexameric l-*Mt*AlaDH molecule. One monomer is shown in the solid ribbon model, and others are shown in gray.

Two forms of structures for l-*Mt*AlaDH have been determined in the Protein Data Bank (PDB), including the dimer (Figure 1C) and the hexamer (Figure 1D) states. The hexameric molecule is made of three-fold non-crystallographic axes, and two monomers nearby form the fold axis. As shown in Figure 1A, each monomer of l-*Mt*AlaDH is composed of a substrate-binding domain (Residues 1–128 and 309–371) and a NAD-binding domain (Residues 129–308), which are connected by two α-helixes [9]. The NAD-binding domain includes a central seven-stranded β-sheet, as well as several surrounding α-helices. The substrate-binding domain consists of an eight-stranded β-sheet and several α-helices [9]. The crystal structure of the open-apo and closed-holo states of the protein have been determined by X-ray crystallography, which can be downloaded from the Protein Data Bank (PDB) with the access codes 2VOE and 2VHZ, respectively [8,9]. The experimental studies have demonstrated that upon the binding of the coenzyme, l-*Mt*AlaDH undergoes a large-scale conformational transition from the open-apo to the closed-holo states, in which the substrate-binding domain (SBD) rotates by 16° toward the NAD-binding domain (NBD) [9]. Considering that the conformational transitions between open and closed structures are essential for the function of l-*Mt*AlaDH, many experimental and theoretical studies have focused on the investigation of the functional domain motions and identification of the related key residues of the protein. The open-closed domain motions of l-*Mt*AlaDH upon the binding of the coenzyme have been studied with molecular dynamics (MD) simulations at the atomic level [10]. The simulation results have shown that the conformational transition of l-*Mt*AlaDH is achieved through the open-closed and twisting motions of the NBD and the SBD. Based on the simulation results, it is proposed that two loops (Residues 94–99 and 238–251) are responsible for the binding of NAD, and another loop (Residues 267–293) is crucially responsible for the binding of substrate [10]. The experiments of Agren *et al.* have shown that l-*Mt*AlaDH completely lost its catalytic activity when the conserved active site residues His96 and Asp270 were mutated. These two residues play an essential role in the enzymatic reaction at the active site and exhibit strong stability in biological evolutionary process [9].

In this work, the domain motions and the related functionally-key residues encoded in the structure of l-*Mt*AlaDH were investigated by using the coarse-grained Gaussian network model (GNM) and the anisotropy elastic network model (ANM) [11,12,13]. The GNM and ANM are simple yet effective methods to explore the intrinsic dynamics of proteins [14,15,16]. It have been proven in numerous application studies that the low-frequency motion modes calculated by GNM and ANM represent the large-scale collective motions usually relevant to the functions of the protein [17,18,19]; whereas the high-frequency motion modes reflect the geometric irregularity in the protein structure, and the residues motivated in these modes are considered to be crucial for the protein stability [20,21]. In the present work, the functional domain motions of l-*Mt*AlaDH were studied through analyzing the slow motion modes of GNM and ANM, and the key residues responsible for the stability of the protein were identified by calculating the fast modes of GNM and ANM. Besides that, the functionally-important residues responsible for substrate binding of the protein were revealed by using a thermodynamic method proposed by Su *et al.* [22,23].

## 2. Results and Discussion

### 2.1. Comparison of the Predicted Mean-Square Fluctuations with the Experimental Temperature Factors (B-Factors)

**Figure 2:**
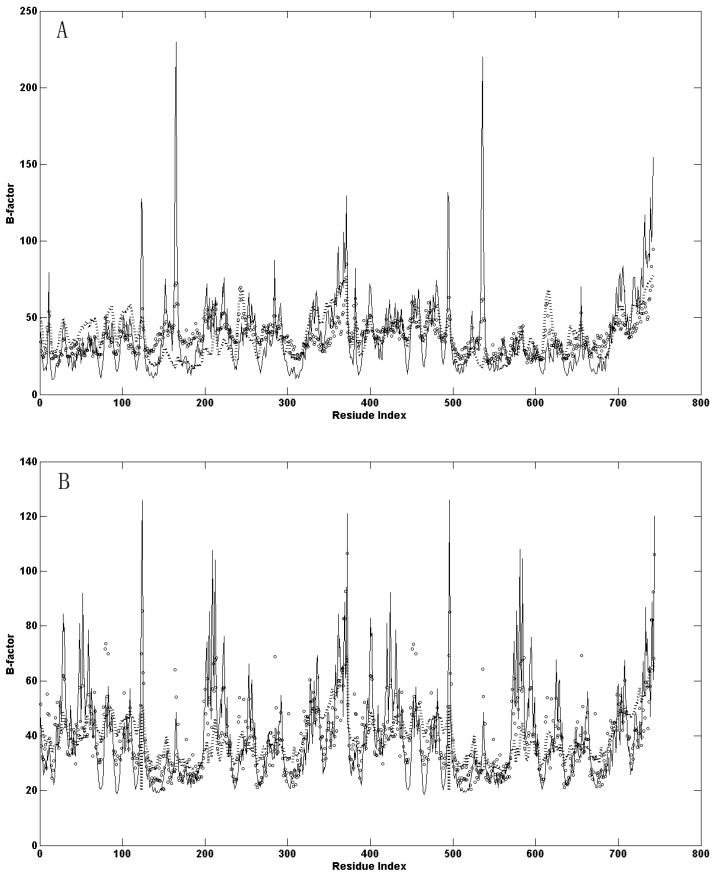
(**A**) The calculated B-factors based on the Gaussian network model (GNM) (circle curve) and the anisotropy elastic network model (ANM) (solid curve) compared to the experimental data (dotted curve) for the open-apo structure of l-*Mt*AlaDH (PDB code: 2OVE); and (**B**) The calculated B-factors based on GNM (circle curve) and ANM (solid curve) compared to the experimental data (dotted curve) for the closed-holo structure of l-*Mt*AlaDH (PDB code: 2VHZ).

According to Equation (7), the B-factors of the residues in the open-apo and closed-holo structures are calculated. In Equation (7), the only adjustable parameter is γ, which is determined by normalizing the computational B-factors to the experimental data. The resulting $k_{B}T/\gamma$ values used for the open-apo and closed-holo structures in the GNM are 3.68 and 6.90 Å^2^, respectively. The resulting $k_{B}T/\gamma$ values used for the open-apo and closed-holo structures in the ANM are 0.45 and 11.64 Å^2^, respectively. Figure 2 shows the comparison between the calculated and the experimental B-factors for the two studied systems. It is found that for GNM, the correlation coefficient between the calculated and experimental Temperature Factors (B-Factors) is 0.50 for the open-apo structure and 0.63 for the closed-holo structure, respectively. For ANM, the correlation coefficients are 0.49 and 0.51 for the open-apo and closed-holo structures, respectively. The correlation coefficient for the two proteins studied in this work is similar to that for other protein [12,24], which provides the validity for the study of *Mt*AlaDH by using the GNM and the ANM.

### 2.2. The Slow Motion Modes

**Figure 3:**
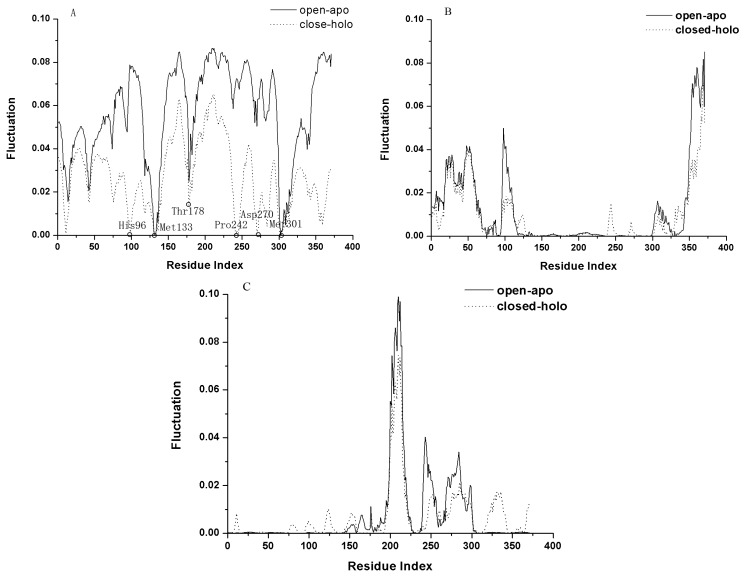
The first three slowest modes of the open-apo (solid line) and the closed-holo (dotted line) structures of l-*Mt*AlaDH are shown in (**A**–**C**), respectively. In (**A**), the important residues located in the minima of the fluctuation curve are marked by circles.

In the GNM, the slow motion modes represent the large-scale collective motions, which are usually relevant to the functions of the protein [25]. Figure 3A shows the first slowest mode calculated by GNM for the open-apo and closed-holo structures of l-*Mt*AlaDH. From Figure 3A, it is found that the first modes for the two studied proteins have a common hinge axes located in Met133 and Met301, with their fluctuation values equal to zero. The fluctuation profiles in Figure 3A also exhibit that the whole structure of the protein is divided into two distinct domains with large fluctuations. Comparing to the slowest mode of the open-apo structure, it can be found that the fluctuation of some residues around the residue His96 is reduced remarkably in the closed-holo structure. This is consistent with the simulation results that the root mean-square fluctuation (RMSF) of the residues around the residue His96 becomes small in the closed state of the protein [10]. It is also found that the fluctuation value of the residues around Asp270 and Pro242 is also approximate to zero, although those residues do not belong to the hinge region (Residues 126–133, 304–320) [10]. These two residues are located at the ligand-binding cleft. This result indicates that the ligand-binding cleft become stable in the closed structure of the protein. Besides the two residues, the residues Met133, Thr178 and Met301 of the NAD-binding domain in the closed-holo structure are located in the minima of the fluctuation curve in Figure 3A. These residues are the ligand-binding sites and are locate in the jaws of the ligand-binding pocket [8]. The crystal structure of the closed-holo state of the protein exhibits that the nicotinamide ring of the ligand is bound in the residues Met133, Ser134, Ala137, Ile267, Asp270 and Met301, and the pyrophosphate moiety forms contacts with the main-chain atoms of residues 178 and 179 [9]. The decrease of the fluctuation of these residues indicates that they are more stable in the closed-holo structure than the open-apo structure. Figure 3B,C show the second and third slowest modes calculated by the GNM for the open-apo and closed-holo structures of l-*Mt*AlaDH, respectively. The overlap between the open-apo and closed-holo structure of the second and third slowest modes is better than the first mode. In Figure 3B,C, the amplitude of the open-apo and close-holo structure is similar. The large fluctuation of the second slowest mode lies on the C-terminal (Residues 342–371) and N-terminal (Residues 21–63) of the protein, and that of the third slowest mode lies in the middle domain of the protein.

It should be noted that besides the structures studied in this work, there are seven other structures of this protein that have been deposited in PDB. The first three slowest modes of these structures of this protein have also been calculated by using the GNM (data not shown). The mode shapes of these structures are found to be similar to those of the open-apo or closed-holo structures studied in this work, which indicates that the functional motions are largely determined by the structural topology of the protein instead of the detailed local structures.

**Figure 4:**
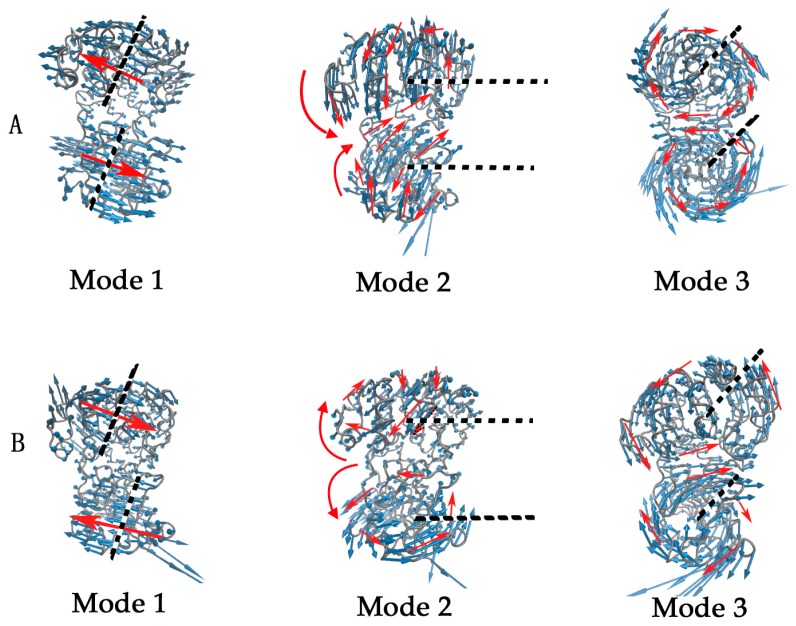
The first three slowest motion modes for the open-apo (**A**) and closed-holo (**B**) structure of l-*Mt*AlaDH. These figures were generated by visual molecular dynamics (VMD) [26]. In this figure, the protein structures are displayed in gray tubes. The amplitude and direction of the motions are represented by the length and direction of the blue arrows, respectively. The red arrows and black dashed lines denote the motion direction and the motion axis, respectively.

GNM can only describe the amplitude of residue fluctuations, but cannot give the direction of the fluctuations. In order to reveal the direction of the motions, ANM is used for the open-apo and closed-holo structures. The first three slowest normal modes calculated by ANM are shown in Figure 4A,B for the open-apo and the closed-holo structures of l-*Mt*AlaDH, respectively. In Figure 4, the amplitude and direction of the motions are represented by the length and direction of the blue arrows, respectively. The red arrows and black dashed lines denote the motion direction and the motion axis, respectively. The first slowest mode (Figure 4 (Mode 1)) of the two structures exhibits a twist motion of the two domains in the proteins. In this motion, the NAD-binding domain (NBD) and the substrate-binding domain (SBD) rotate reversely around their respective axes. The rotation axes are displayed in Figure 4 (Mode 1). This motion mode might be important for the two domains in the proteins to adjust their relative position to bind or dissociate the ligand. The long blue arrows represent the amplitude of the motions. This is consistent with the first slowest mode of GNM. The second slowest mode describes a hinge-bending motion of the NBD and SBD for the two studied proteins, as shown in Figure 4 (Mode 2). The α-helixes connecting the NBD and SBD serve as the hinge of the bending motion. The motion results in the opening and closing of the ligand-binding pocket. The studies by MD simulations also showed that the SBD rotates about 14.2° around the hinge axis toward the NBD of the protein [10]. This is consistent with the second slowest motion mode of GNM, where the fluctuation of the C-terminus is larger than other residues. The third slowest mode (Figure 4 (Mode 3)) represents a reverse rotation of the NBD and SBD around their respective axes. Additionally, the rotation axes of these two domains are marked by black dashed lines in this figure.

**Figure 5:**
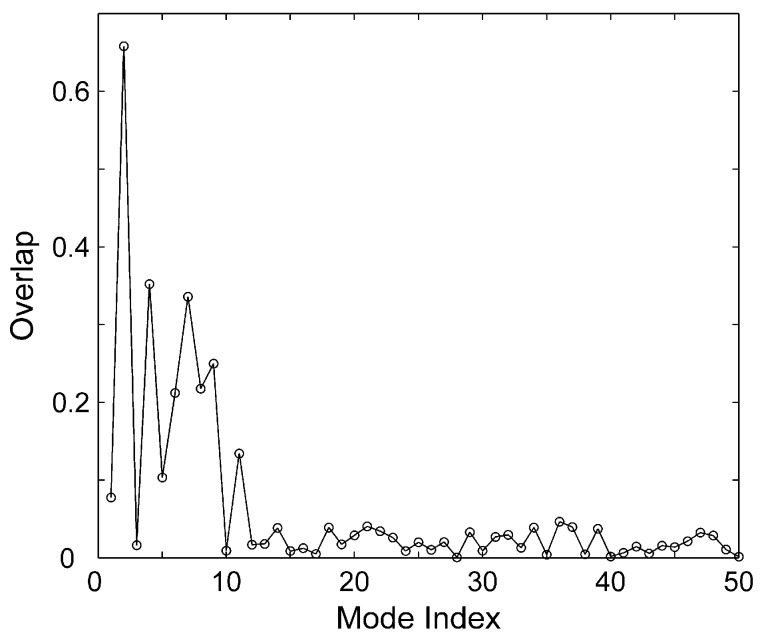
The overlap between the first 50 modes and the conformational change.

In order to investigate whether the normal modes can reveal the conformational change observed by the experiment, the overlap between each mode and the experimental conformational change was calculated using Equation (17). Figure 5 shows the overlap for the first 50 modes. It is found that Mode 2 mostly contributes to the conformational change, where the value of the overlap is 0.658.

**Figure 6:**
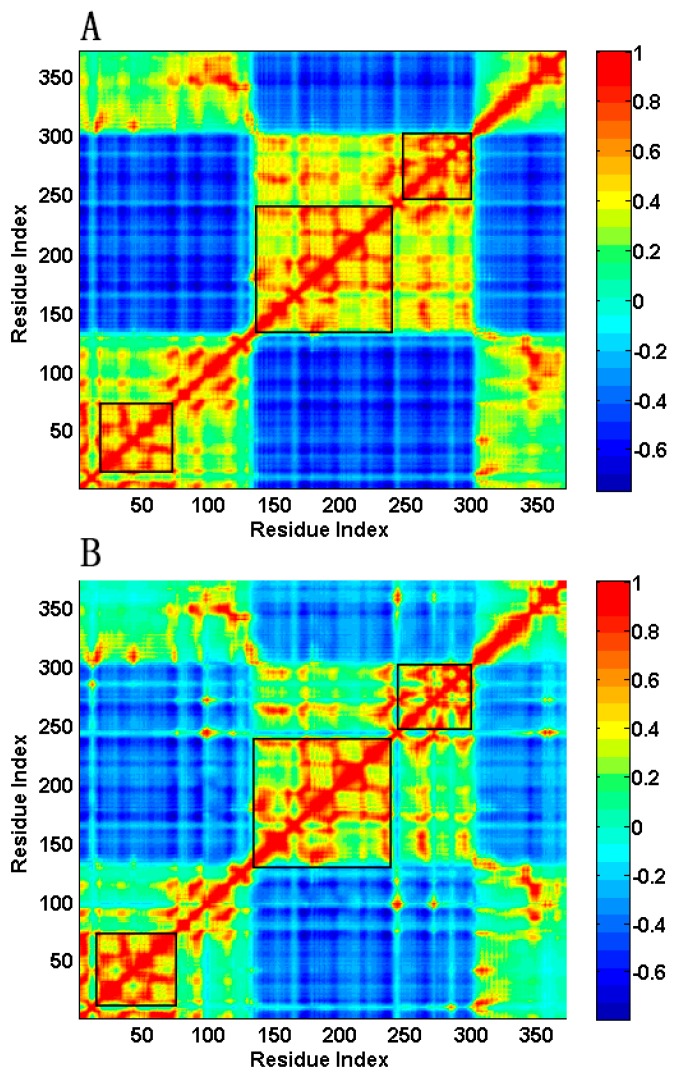
The cross-correlation maps calculated by the first 30 slowest modes of GNM for the open-apo (**A**) and the closed-holo (**B**) structures of l-*Mt*AlaDH. In this figure, blue regions indicate negative correlation and orange-red regions indicate positive correlation, as shown in the color bar on the right. The regions in the rectangle (Residues 16–74, 134–241, 243–300) indicate the highly coupled regions.

In order to analyze the coupling between the residues motions, the cross-correlation between residue fluctuations is calculated using Equation (10). Because the low-frequency normal modes are usually relevant to protein functions, the first 30 slowest modes are considered in the calculation. The calculation results are displayed in Figure 6. The value of the cross-correlation ranges from −1 to 1, where the positive value represents the residues moving in the same direction and the negative value means the residues moving in the opposite direction. In Figure 6, the regions in orange-red color represent positive correlation and the blue regions denote negative correlation. It is found that the cross-correlation map, both for the open-apo and closed-holo structures, is divided into five orange regions and four blue regions separated by the residues Met133 and Met301, which means that these two residues serve as the hinges for the correlated motions. This is consistent with the result obtained by the analysis of the slowest normal mode, in which these two residues act as the motion hinges with almost zero fluctuations. In Figure 6, the blue regions correspond to the negative correlation between the motions of NBD and SBD, illustrating the open-closed conformational transition of the protein. The orange-red region in the center of the maps (Residues 133–301) represents the residues of the NBD moving as a whole, whereas the other four orange-red regions indicate the residues of the SBD also moving in a coupled way. This result indicates that the two domains keep their rigid structure during the conformational transitions. Comparing the closed-holo structure to the open-apo structure of the protein, more contacts are formed for the residues in the jaws of the ligand-binding pocket. These residue contacts may reduce the correlation between domain motions. Therefore, the blue and orange-red colors are weakened in the correlation map for the closed-holo structure compared to that for the open-apo structure, as shown in Figure 6. From Figure 6B, it is found that the fluctuation correlations among the residues Arg15, Loops L1 (Residues 94–99), L2 (Residues 238–247) and L3 (Residues 267–270) are significantly improved in the closed-holo structure because of their interactions with the ligand. In Figure 6, there are several regions with high correlation values, marked by the rectangles. These regions correspond to the highly coupled movement of residues 134–241 and 243–300 of NBD and residues 16–74 of SBD.

### 2.3. The Fast Motion Modes

**Figure 7:**
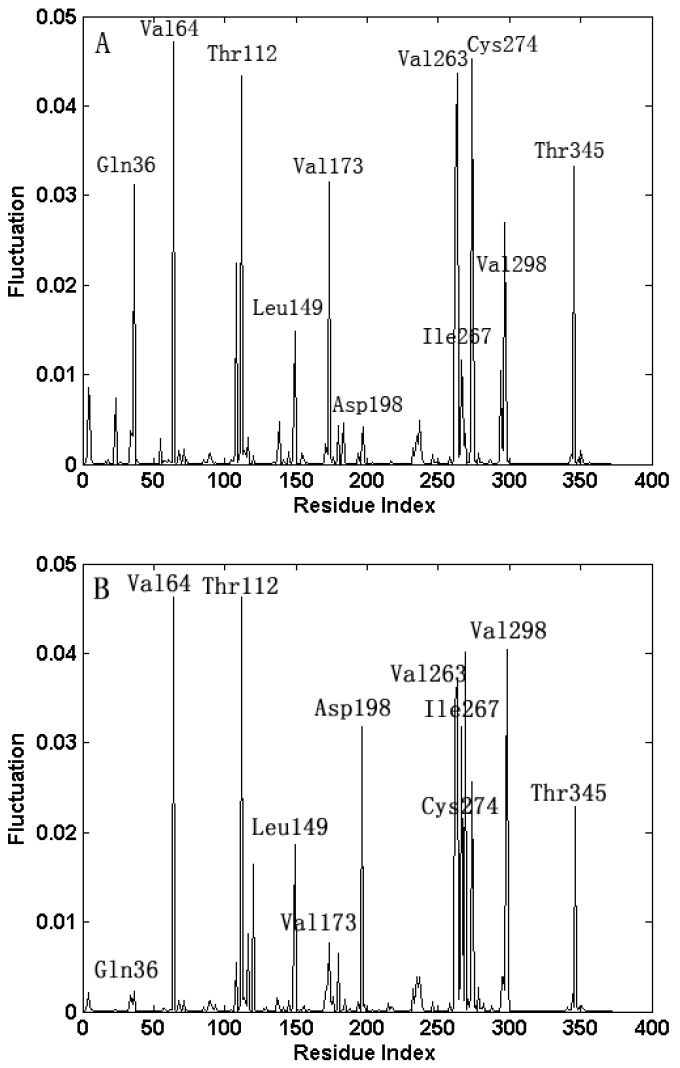
(**A**,**B**) The mode shape as the average of the eight fastest modes for the open-apo and closed-holo structure of l-*Mt*AlaDH. The same key residue clusters identified by our method are marked by the residue name in the figure.

In GNM, the fast motion modes reflect the geometric irregularity in the local structure, and the fluctuations in the fast motion modes are accompanied with the remarkable increases of the entropy. Then, the residues activated in the fast motion modes are regarded as the key residues that are important for the stability of the protein structure [21,25]. Figure 7 shows the eight fastest modes for the open-apo and closed-holo structures, respectively. It is found that the mode shapes for the open-apo and closed-holo structures are extremely similar, and the residues at the peaks of the fluctuation are almost the same, implying that the same set of residues is responsible for the stability of the open-apo and closed-holo structures of the protein. This result implies that the local structures are similar for these two proteins, and the domains of the protein keep their rigid structures during the open-closed motions. Figure 7 shows that the hot residues responsible for the stability of the proteins include Leu149, Val173, Asp198, Val263, Ile267, Cys274 and Val298 in NBD and Gln36, Val64, Thr112 and Thr345 in SBD. From the crystal structure of the proteins, it is found that these key residues are tightly packed, and most of them lie in the ligand-binding pocket, among which the residues Asp198 and Ile267 form polar interactions with the ligand NAD [8]. Our results imply that the residues crucial for protein stability also play important roles for the binding of the substrate to the protein.

### 2.4. The Functionally-Important Residues Identified with the Thermodynamic Method

**Figure 8:**
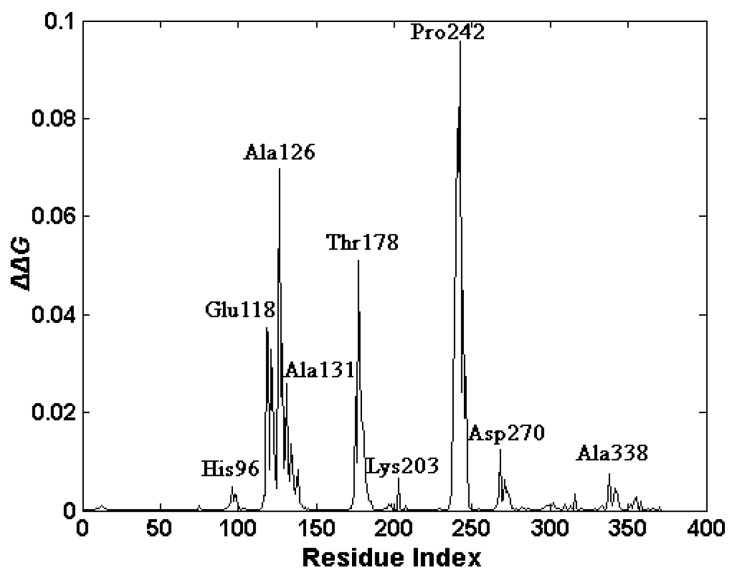
ΔΔ*G* values in response to residual perturbations. The residues of the clusters with peak values are marked.

In our work, the functionally-key residues responsible for the ligand binding were identified by using a thermodynamic method proposed by Su *et al.* [22,23,27]. The closed-holo structure of the protein is adopted as the complex structure, and the corresponding GNM was constructed. Then, the ligand NAD was removed from the closed-holo structure, and the corresponding GNM was also constructed. Based on these two constructed models, each residue of the protein was perturbed, and the ΔΔ*G* value in response to the perturbation was calculated according to the method proposed in the Methods Section. In our method, the free energy change ∆∆*G* for each residue is the sum of the free energy changes for all of the spring perturbations involved in this residue. Residues with relatively high ΔΔ*G* values were regarded as the key residues involved in the ligand binding of the protein. The calculated result is shown in Figure 8. It was found that nine clusters of residues exhibit high ΔΔ*G* values, which are centered at the residues His96, Glu118, Ala126, Met133, Thr178, Lys203, Pro242, Asp270 and Ala338, respectively.

From Figure 8, it is found that almost all of these key residues are located at the ligand-binding site in the NBD. Tripathi *et al.* have proposed that NAD interacts extensively with enzyme through the interactions with Met301, Asp270, Ile267, Thr178, Lys203, Asp198, Met133, His96, Lys75 and Arg15 [8]. Most of our predicted residues belong to the residues directly interacting with NAD. The analysis of the slowest normal mode discussed in the previous section has identified Met133 to be the motion hinge. This residue plays an important role for the opening and closing of the ligand-binding pocket, and thus, it is crucial for the binding of the ligand to the protein. The residues His96 and Asp270 were identified as the conserved active site residues by Agren’s group [9]. The residue Ala126 is also located in the hinge region between the two domains of the protein. In the slowest motion mode of the closed structure of the protein, the residues Thr178, Pro242 exhibit small fluctuations, which imply that these residues play important roles in the binding of the ligand, stabilizing the closed-holo structure of l-*Mt*AlaDH. The functionally-key residues predicted by the thermodynamic method are consistent with the experimental observations and the results obtained by the analysis of the slow modes.

## 3. Materials and Methods

### 3.1. The Gaussian Network Model and the Anisotropy Elastic Network Model

In the Gaussian network model (GNM) [11,28], the tertiary structure of a protein is simplified as an elastic network. Each residue of the protein is represented by a point that is located at its C_α_ atom, and all of the pairwise residues within a cutoff distance (8 Å is adopted here) are connected by a harmonic springs with a uniform force constant. Then, the internal energy of the protein can be expressed as [15]:

(2) $$H=\frac{1}{2}\gamma \left[\Delta R^{T}(\Gamma ⊗E)\Delta R\right]$$

where γ is the spring constant; ΔR represents fluctuations of the residues; *E* is the identity matrix; ⊗ is the direct product of the matrix; *T* represents the transpose of the matrix; Γ is the Kirchhoff matrix with N×N elements that can be expressed as [11,15]:

(3) $$\Gamma =\left\{ \begin{array}{llll} -1, & if\;i\neq j & and & R_{ij}\leq r_{c}\\ 0, & if\;i\neq j & and & R_{ij}>r_{c}\\ -\sum_{i,i\neq j}\Gamma_{ij}, & if\;i=j \end{array}\right.$$

where $R_{ij}$ is the separation between the *i*-th and *j*-th $C_{\alpha }$ atoms and $r_{c}$ is the cutoff distance.

The cross-correlation between the fluctuations of the *i*-th and *j*-th residues is written as:

(4) $$\langle \Delta R_{i}\cdot \Delta R_{j}\rangle =\frac{3k_{B}T}{\gamma }{\left[\Gamma^{-1}\right]}_{ij}$$

When *i* = *j*, the mean-square fluctuation of the *i*-th residue can be written as:

(5) $$\langle {\left(\Delta R_{i}\right)}^{2}\rangle =\frac{3k_{B}T}{\gamma }{\left[\Gamma^{-1}\right]}_{ii}$$

where $k_{B}$ is the Boltzmann constant; T is absolute temperature.

The inverse of the Kirchhoff matrix can be calculated by diagonal decomposition as:

(6) $$\Gamma^{-1}=U\Lambda^{-1}U^{T}$$

where U is an orthogonal matrix whose columns represent the eigenvectors of Γ; Λ is a diagonal matrix, and the diagonal elements are the eigenvalues of Γ.

According to the Debye-Waller theory, the B-factor of the *i*-th residue can be calculated by:

(7) $$B_{i}=8\pi^{2}\langle \Delta R_{i}\cdot \Delta R_{i}\rangle /3$$

The mean-square fluctuation of the *i*-th residue in h the *k*-th mode is written by:

(8) $${\langle \Delta R_{i}\cdot \Delta R_{i}\rangle }_{k}=\frac{3k_{B}T}{\gamma }\lambda_{k}^{-1}{\left[u_{k}\right]}_{i}{\left[u_{k}\right]}_{i}$$

The cross-correlation between the fluctuations of different residues is written by:

(9) $$\langle \Delta R_{i}\cdot \Delta R_{j}\rangle =\frac{3k_{B}T}{\gamma }\sum_{k=2}^{N}\lambda_{k}^{-1}{\left[u_{k}\right]}_{i}{\left[u_{k}\right]}_{j}$$

In the GNM, the cross-correlation is normalized as:

(10) $$C_{ij}=\frac{\langle \Delta R_{i}\cdot \Delta R_{j}\rangle }{{\left[\langle \Delta R_{i}^{2}\rangle \times \langle \Delta R_{j}^{2}\rangle \right]}^{1/2}}$$

For ANM, the motion mode of the protein is determined by the 3N×3N Hessian matrix *H*.

(11) $$H=\left(\begin{array}{cccc} h_{11} & h_{12} & \ldots & h_{1N}\\ h_{21} & h_{22} & \ldots & h_{2N}\\ \vdots & \vdots & \vdots & \vdots \\ h_{N1} & h_{N2} & \ldots & h_{NN} \end{array}\right)$$

The elements of *H* are a submatrix with a size of 3×3. The *ij*-th submatrix $h_{ij}$ is:

(12) $$h_{ij}=\left(\begin{array}{ccc} \frac{\partial^{2}V}{\partial x_{i}\partial x_{j}} & \frac{\partial^{2}V}{\partial x_{i}\partial y_{j}} & \frac{\partial^{2}V}{\partial x_{i}\partial z_{j}}\\ \frac{\partial^{2}V}{\partial y_{i}\partial x_{j}} & \frac{\partial^{2}V}{\partial y_{i}\partial y_{j}} & \frac{\partial^{2}V}{\partial y_{i}\partial z_{j}}\\ \frac{\partial^{2}V}{\partial z_{i}\partial x_{j}} & \frac{\partial^{2}V}{\partial z_{i}\partial y_{j}} & \frac{\partial^{2}V}{\partial z_{i}\partial z_{j}} \end{array}\right)$$

When i=j,

(13) $$h_{ij}=\left(\begin{array}{ccc} \frac{\partial^{2}V}{\partial x_{i}^{2}} & \frac{\partial^{2}V}{\partial x_{i}\partial y_{i}} & \frac{\partial^{2}V}{\partial x_{i}\partial z_{i}}\\ \frac{\partial^{2}V}{\partial y_{i}\partial x_{i}} & \frac{\partial^{2}V}{\partial y_{i}^{2}} & \frac{\partial^{2}V}{\partial y_{i}\partial z_{i}}\\ \frac{\partial^{2}V}{\partial z_{i}\partial x_{i}} & \frac{\partial^{2}V}{\partial z_{i}\partial y_{i}} & \frac{\partial^{2}V}{\partial z_{i}^{2}} \end{array}\right)$$

where the elements $\frac{\partial^{2}V}{\partial x_{i}\partial y_{j}}$, $\frac{\partial^{2}V}{\partial x_{i}\partial y_{i}}$ of the submatrix $h_{ij}$ can be analytically expressed as:

(14) $$\frac{\partial^{2}V}{\partial x_{i}\partial y_{j}}={\frac{-\gamma (x_{j}-x_{i})(y_{j}-y_{i})}{r_{ij}^{2}}|}_{r_{ij}=r_{ij}^{0}}$$

(15) $$\frac{\partial^{2}V}{\partial x_{i}\partial y_{i}}={\gamma \sum_{j\neq i}\frac{\left(x_{j}-x_{i})(y_{j}-y_{i}\right)}{r_{ij}^{2}}|}_{r_{ij}=r_{ij}^{0}}$$

where *x*, *y* and *z* represent the coordinates of each atom.

### 3.2. Correlation Coefficient and Overlap

In our work, the correlation coefficient between the calculated and the experimental B-factors is written as:

(16) $$\rho =\frac{\sum_{i=1}^{N}\left(x_{i}-\bar{x})(y_{i}-\bar{y}\right)}{{\left[\sum_{i=1}^{N}{\left(x_{i}-\bar{x}\right)}^{2}\sum_{i=1}^{N}{\left(y_{i}-\bar{y}\right)}^{2}\right]}^{1/2}}$$

where $x_{i}$ and $y_{i}$ are the calculated and experimental values for the B-factor of the *i*-th $C_{\alpha }$ atom, $\bar{x}$ and $\bar{y}$ are the mean values of $x_{i}$ and $y_{i}$ and *N* is the total number of $C_{\alpha }$ atoms in the protein.

Because the open-apo and closed-holo structures of the protein have been determined by the X-ray crystallographic experiment, the conformational change can be calculated. Then, we investigated whether the slow motion modes obtained by ANM can account for the experimental conformational change, which can be evaluated by the overlap between the normal modes and the conformational change. The value of overlap can be calculated by:

(17) $$overlap_{i}=\frac{\vec{\eta_{i}}\cdot \Delta {\vec{R}}_{\exp}}{\left|\Delta {\vec{R}}_{\exp}\right|}$$

where $\vec{\eta_{i}}$ is the *i*-th normal mode, $\Delta {\vec{R}}_{\exp}$ is the experimental conformational change and $\left|\Delta {\vec{R}}_{\exp}\right|$ is the norm of $\Delta {\vec{R}}_{\exp}$.

### 3.3. Thermodynamic Cycle Method

Base on the elastic network model (ENM), a thermodynamic method was developed by our group to reveal the functionally-important residues involved in the binding of ligand [22,23,27]. According to the theory of statistical physics, the vibrational entropy of ENM can be expressed as [25]:

(18) $$S=\frac{\langle H\rangle -F}{T}=\frac{1}{T}\left[\frac{3}{2}\left(N-1\right)k_{B}T+k_{B}TInZ\right]=\frac{3}{2}\left(N-1\right)k_{B}+k_{B}InZ$$

where 〈H〉 is the average value of the Hamiltonian; $F=-k_{B}TInZ$ is the Helmholtz free energy; T is the temperature; N is the number of residues in the protein; $k_{B}$ is the Boltzmann constant; Z is the configurational integral part of the vibrational partition function that can be calculated by $Z=\int \exp(-H/k_{B}T)d\left\{\Delta R\right\}$; {ΔR} represents the 3N-dimensional column vector of fluctuations. When a perturbation of the spring between the *i*-th and *j*-th residues is introduced, the vibrational entropy of the protein will be changed as:

(19) $$\Delta S=-\frac{\partial S}{\partial \gamma_{ij}}\Delta \gamma_{ij}$$

where Δγ_ij_ is the change of the force constant that accounts for the perturbation of the spring. According to Equation (18), the derivation of the entropy with respect to γ_ij_ can be expressed as [22,23]:

(20) $$\frac{\partial S}{\partial \gamma_{ij}}=-\frac{1}{2T}\left(\langle {\left(\Delta R_{i}\right)}^{2}\rangle +\langle {\left(\Delta R_{j}\right)}^{2}\rangle -2\langle {\left(\Delta R_{i}\cdot \Delta R_{j}\right)}^{2}\rangle \right)$$

In this method, all of the residues in the protein were perturbed one by one, and then, the change of the free energy for the protein-ligand binding was calculated.

**Figure 9:**
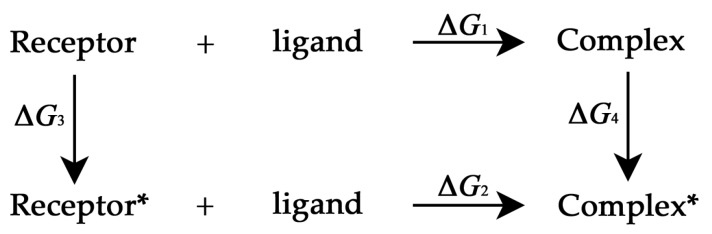
The thermodynamic cycle, Δ*G*_1_ denotes the ligand-binding free energy without the introduction of the perturbation, and Δ*G*_2_ denotes the value of the binding free energy with a residue perturbation. The receptor* and complex* represent the systems where a residue perturbation is introduced. Two nonphysical processes were constructed, *i.e.*, Receptor → Receptor* and Complex → Complex*. The free energy changes associated with these two constructed processes are denoted as Δ*G*_3_ and Δ*G*_4_.

In Figure 9, Δ*G*_1_ denotes the ligand-binding free energy without the introduction of the perturbation, and Δ*G*_2_ denotes the value of the binding free energy with a residue perturbation. In this work, the binding ligand is 1,4-dihydronicotinamide adenine dinucleotide (NAD). The receptor* and complex* represent the systems where a residue perturbation is introduced. We want to calculate the change of the free energy resulting from the spring perturbation, *i.e.*,

(21) $$\Delta \Delta G=\Delta G_{2}-\Delta G_{1}$$

The residues with relatively large ΔΔ*G* values are identified as the functionally-important residues, which are thermodynamically coupled with the binding of ligand to the protein. In order to calculate the free energy changes more easily, a thermodynamic cycle was proposed. Two nonphysical processes were constructed, *i.e.*, Receptor → Receptor* and Complex → Complex*. The free energy changes associated with these two constructed processes are denoted as Δ*G*_3_ and Δ*G*_4_. Then, Equation (21) can be expressed as:

(22) $$\Delta \Delta G=\Delta G_{2}-\Delta G_{1}=\Delta G_{4}-\Delta G_{3}$$

When a perturbation was introduced to a residue of the protein, the force constant of the corresponding spring is reduced, and the perturbation of γ_ij_ results in the same change of the potential energy of Receptor and Complex, *i.e.*, Δ*U*_3_ = Δ*U*_4_. Then:

(23) $$\Delta \Delta G=(\Delta U_{4}-T\Delta S_{4})-(\Delta U_{3}-T\Delta S_{3})=-T(\Delta S_{4}-\Delta S_{3})$$

In our method, when a perturbation was introduced to a residue, all of the springs connecting to the residue should be perturbed in the same way. In this way, the ΔΔ*G* value resulting from the perturbation of a residue is calculated by the sum of the ΔΔ*G* values of all of the perturbed springs involved in this residue. It should be pointed out that only the first 30 slowest normal modes of the closed-holo structure were considered in our calculation, and for the protein-ligand complex, all of the modes were projected onto these slowest modes.

## 4. Conclusions

The open-closed conformational changes of l-*Mt*AlaDH induced by the coenzyme are important for the catalytic function of the protein. In this work, the domain motions and the functionally-key residues encoded in the structure of the protein were investigated by using the coarse-grained Gaussian network model (GNM) and the anisotropy network model (ANM). The calculated B-factors are consistent with the experimental data from the X-ray crystallographic experiments, which provide the validity for the application of the GNM to the studied system l-*Mt*AlaDH. The analysis of the slowest normal mode for the open-apo and the closed-holo structures shows that the two domains in the proteins undergo large-scale motions, where the motion axes are centered at Met133 and Met301. The ANM calculation results indicate that both for the open-apo and the closed-holo structures, the first three slow modes respectively represent the twist, hinge-bending and rotation motions of the proteins. These motions are responsible for the conformational transition of the proteins and contribute to the opening and closing of the ligand binding pocket. The key residues responsible for the stability of the proteins were successfully identified by analyzing the high-frequency normal modes. It is found that the key residues are tightly packed, and most of them lie in the ligand-binding pocket. The results of cross-correlation analysis implied that the residues in two domains of the protein move in a highly coupled way. In addition, the key residues responsible for the ligand binding were identified by using the thermodynamic cycle method. The predicted functionally-key residues are consistent with the experimental observations. Our method is a simple and coarse-grained model, which considered the connections between C_α_ atoms only. Our methods should be combined with other information to determine the functionally-important residues more exactly and should be combined with more novel methods to calculate the interaction of the side chain. We will construct a more detailed model, in which the side chains of residues are explicitly considered, in the next step to improve our method.
